# The Truncate Mutation of Notch2 Enhances Cell Proliferation through Activating the NF-κB Signal Pathway in the Diffuse Large B-Cell Lymphomas

**DOI:** 10.1371/journal.pone.0108747

**Published:** 2014-10-14

**Authors:** Xinxia Zhang, Yaoyao Shi, Yuanyuan Weng, Qian Lai, Taobo Luo, Jing Zhao, Guoping Ren, Wande Li, Hongyang Pan, Yuehai Ke, Wei Zhang, Qiang He, Qingqing Wang, Ren Zhou

**Affiliations:** 1 Department of Pathology and Pathophysiology, Institute of Pathology and Forensic Medicine, Zhejiang University School of Medicine, Hangzhou, China; 2 Institute of Immunology, Zhejiang University School of Medicine, Hangzhou, China; 3 Department of Pathology, the First Affiliated Hospital, Zhejiang University School of Medicine, Hangzhou, China; 4 Department of Biochemistry, Boston University School of Medicine, Boston, Massachusetts, United States of America; 5 Epitomics (Hangzhou) Inc., Hangzhou, China; 6 Zhejiang Province People's Hospital, Hangzhou, China; University of Navarra, Center for Applied Medical Research, Spain

## Abstract

The Notch2 is a critical membrane receptor for B-cell functions, and also displays various biological roles in lymphoma pathogenesis. In this article, we reported that 3 of 69 (4.3%) diffuse large B-cell lymphomas (DLBCLs) exhibited a truncate *NOTCH2* mutation at the nucleotide 7605 (G/A) in the cDNA sequence, which led to partial deletion of the C-terminal of PEST (proline-, glutamic acid-, serine- and threonine-rich) domain. The truncate Notch2 activated both the Notch2 and the NF-κB signals and promoted the proliferation of B-cell lymphoma cell lines, including DLBCL and Burkitt's lymphoma cell lines. Moreover, the ectopic proliferation was completely inhibited by ammonium pyrrolidinedithiocarbamate (PDTC), an NF-κB inhibitor. Simultaneously, PDTC also reduced the expression level of Notch2. Based on these results, we conclude that the Notch2 receptor with PEST domain truncation enhances cell proliferation which may be associated with the activation of the Notch2 and the NF-κB signaling. Our results are expected to provide a possible target for new DLBCL therapies by suppressing the Notch2 and the NF-κB signaling.

## Introduction

**Figure 1:**
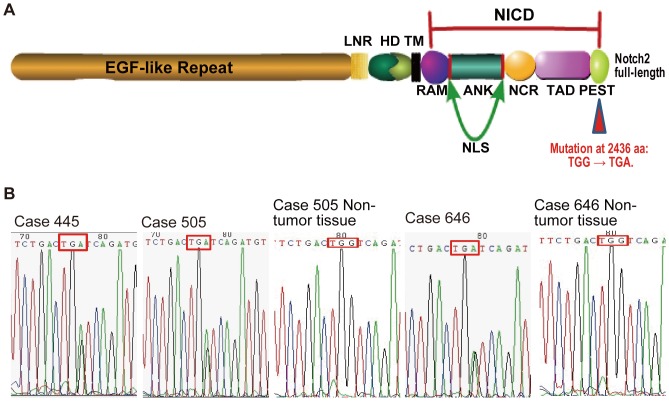
The mutant status of the *NOTCH2*. (A) The extracellular domain of Notch2 contains epidermal growth factor (EGF)-like repeats, three cysteine-rich LIN/Notch repeats (LNR) and heterodimerization domain (HD). The cytoplasmic domain contains RBP-Jκ associated molecule (RAM) domain, six ankyrin/CDC10 repeats (ANK) including two nuclear localization signals (NLS), Notch cytokine response (NCR) domain, transactivation domain (TAD) and PEST (proline-, glutamic acid-, serine- and threonine-rich) domain. There is a transmembrane domain (TM) between extracellular domain and cytoplasmic domain. The identical mutation of the three DLBCLs located at 2436^th^ amino acid in the PEST domain, which converted a tryptophan (TGG) into a termination codon (TGA), and resulted in a truncate Notch2. (B) The identical mutation of the three DLBCLs was at the nucleotide 7605 (G/A) in the human *NOTCH2* cDNA sequence. In the matched surrounding non-tumor tissues of both No. 505 and No. 646, the 7605 site showed “G” which was identical to the germline sequences; but matched non-tumor tissue was not available in No. 445.

The *NOTCH* gene was first described by Morgan in *Drosophila melanogaster* in 1917 [1]. In mammals, four Notch receptors (Notch1 to 4) and five Notch ligands are reported. The Notch proteins are single-pass transmembrane receptors (Fig. 1A): the extracellular domain is related to binding the Notch ligands, and the Notch intracellular domain (NICD, a transcription nuclear factor) is responsible for transferring the Notch signal into the nucleus [2], [3] and activating the Notch signal pathway [4]. The Notch signaling has many important biological functions, including cell proliferation, apoptosis, stem cell maintenance [5], lymphocyte activation and differentiation [6]–[8]. Furthermore, Notch2 is a crucial receptor for B-cell functions [5]. Recently, many reports have shown that there may be a cross-talk between the Notch and the NF-κB signaling [9]–[12]. The Notch2 can affect the expression of the NF-κB, and the NF-κB signaling can also regulate the expression of Notch2 or components of the Notch2 pathway [13]. The NF-κB is an important regulator for multiple cellular activities such as proliferation, differentiation and survival [14]. In mammals, the NF-κB family embraces the following members: p65 (RelA), RelB, c-Rel, p50/p105 (NF-κB1) and p52/p100 (NF-κB2) [15]. The proteasome-mediated degradation of IκBα (a member of IκB family) results in the release of active NF-κB, and finally activates the NF-κB signal pathway [16]. During the activation processes, the phosphorylation of NF-κB members plays a crucial role.

The Notch2 receptor is highly conserved from invertebrates to vertebrates. The mutations of *NOTCH2* are very rare and cluster in the heterodimerization (HD) domain and the PEST domain (Fig. 1A), which can bind E3 ubiquitin ligases and induce the PEST domain-dependent NICD degradation [17]. Recently, some *NOTCH2* mutations have been detected in some B-cell non-Hodgkin's lymphomas (B-NHLs) subtypes, such as DLBCLs [18] and marginal zone lymphomas (MZLs) [19], especially splenic marginal zone lymphomas (SMZLs, one subtype of MZLs) [20], [21]. *NOTCH2* mutations identified in DLBCLs are thought to activate the Notch2 signaling [18]. DLBCL is the most common type of B-NHLs [22], and it is well known to be highly heterogeneous both histologically and clinically. Through gene expression profiling, DLBCLs can be divided into at least three subgroups: germinal center B-cell like (GCB) DLBCLs, activated B-cell like (ABC) DLBCLs, and primary mediastinal large B-cell lymphomas [23], [24]. Current studies focus on the algorithm and therapeutic strategy of DLBCLs, which heavily relied on the researches of the important oncogenes such as *NOTCH2*. It is still a puzzle that whether a mutation in *NOTCH2* is a primary or secondary event in the evolution of DLBCLs.

In this study, we performed DNA sequencing of the *NOTCH2* gene in human DLBCLs. We found a truncate mutation in the PEST domain of the *NOTCH2* gene in several DLBCL cases. This recurrent mutation promoted cell proliferation may through activation of both the Notch2 and the NF-κB signals.

## Materials and Methods

### Patients and Genomic DNA Preparation

A total of 115 B-NHLs paraffin-embedded samples (Table S1) were collected from the affiliated hospitals of Zhejiang University after obtaining written informed consents; and some of them had paired surrounding non-tumor tissues. In each case, diagnosis of B-NHL was made according to the World Health Organization Classification of Tumors of Hematopoietic and Lymphoid Tissues [22] by at least two senior local diagnostic pathologists. This study was approved by the Ethics Committee of Zhejiang University (Hangzhou, China). Genomic DNA was extracted from the paraffin-embedded tissues using the phenol chloroform method.

### Cell Culture

293T and Burkitt's lymphoma cell lines (Raji, Daudi and Namalwa) were obtained from the Type Culture Collection of the Chinese Academy of Sciences (Shanghai, China); Ramos (Burkitt's lymphoma cell line) and Pfeiffer (DLBCL cell line) cell lines were purchased from the ATCC; and OCI-ly3 (DLBCL cell line) and OCI-ly6 (DLBCL cell line) cell lines were gifted by Dr. X. Jiang (China) and Professor T. Zhao (China), respectively. The 293T cell line was maintained in Dulbecco's modified Eagle's medium with 10% FBS (Gibco). Most lymphoma cell lines were maintained in Roswell Park Memorial Institute-1640 (RPMI-1640) with 10% FBS (Gibco), and the OCI-ly3 and the OCI-ly6 cell lines were maintained in ISCOVE's modified DMEM (IMDM) with 10% FBS (Gibco).

### Mutation Analysis of *NOTCH2*

DNA extracted from the 115 B-NHLs cases was analyzed for the *NOTCH2* mutation on exon 26 (HD domain) and exon 34 (PEST domain and its bilateral flanking regions) by PCR as described [25]. After direct sequencing of both strands, purified PCR products were compared with germline sequences and germline polymorphisms available in the NCBI SNP database (http://www.ncbi.nlm.nih.gov/projects/SNP/).

### Immunohistochemistry

The immunohistochemical staining of DLBCLs was performed according to the protocol [18]. The immunohistochemistrical antibodies were listed as follows: CD10 (56C6, Dako), Bcl6 (M7211, Dako), MUM1 (MUM1p, Dako), Ki67 (MB1, Dako), P50 (sc-8414, Santa Cruz), P65 (#4764, Cell Signaling) and Notch2 (ab52302, Abcam). For Ki67 [26] and Notch2 [27], cases were considered to be positive when 25% or 20% of lymphoma cells were stained, respectively. For the rest antibodies, we defined 30% as the cut-off [28], [29].

### Plasmid Construction

The human NICD (5392–7710 bp, NM_024408.3; 1699–2470 aa, NP_077719.2) [30] amplified from 293T cell cDNA and the six-repeating myc tag were constructed into the pLVX-Puro vector (PT4002-5, Clontech Laboratories). The recombined Notch2-pLVX-Puro vector was used as the wild-type Notch2 expression vector (wt Notch2). Based on the *NOTCH2* mutation analysis and the wt Notch2 vector, a mutant-type Notch2 expression vector (mt Notch2, 5392–7602 bp, NM_024408.3; 1699–2435 aa, NP_077719.2) was constructed. The human *RBP-Jκ* (recombination signal binding protein for immunoglobulin kappa J region) full-length (237–1736 bp, NM_005349.3; 1–499 aa, NP_005340.2) cDNA was cloned into the pcDNA3.1(-)2HA vector. The −194 to +60 promoter fragment of the *HES-1* gene was cloned upstream of the luciferase gene in the pGL3 luciferase vector (E1751, Promega) as the HES-1 luciferase reporter [31].

### Transfections and Infections

Transfections or infections with either wt Notch2, mt Notch2 or empty vector (pLVX) were carried out as described [32]. 293T cells were co-transfected with different recombinant along with helper plasmids (psPAX2 and pMD2.G). Virus supernatants were collected at 24 h, 48 h and 72 h post-transfection, and then were precipitated, concentrated and re-suspended. After measurements of transduction unit titer, the virus was used to infect lymphoma cells (Daudi, Raji, Ramos, Namalwa, Pfeiffer, OCI-ly3 and OCI-ly6). Stably-infected cell lines were obtained after selection by long-term culture in medium containing 2 µg/mL puromycin (Invitrogen) for 30 days.

### Real-time PCR

After 24 h infection, total RNA was isolated by Trizol (Invitrogen) and 1 µg RNA reverse-transcribed using a PrimeScript RT reagent kit (Takara). Real-time PCR was performed with SYBR Premix Ex TaqTM (Takara), using the 7500 Real-Time PCR System (Applied Biosystems). The real-time primer sequences were listed in Table S2. Relative fold change was normalized to GAPDH and calculated with the 2^−ΔΔct^ method. Each experiment was performed independently for three times at least.

### Luciferase Reporter Assay

The Notch2 (wt or mt, 4 µg) or the pLVX vector (4 µg) was co-transfected with the pRL-TK vector (50 ng, Promega) and luciferase reporter plasmid, such as 12× RBP-Jκ (2 µg, a gift from U. Lendahl, Sweden), Hes1 (2 µg) or NF-κB (2 µg, Beyotime), into 2×10^6^ lymphoma cells by electro-transfection (Nucleofector II, Amaxa Biosystems) using the Amaxa Cell Line Nucleofector Kit V (Lonza). After 12–24 h, a Dual-Luciferase Reporter Assay System (Promega) was used to detect luciferase activities. Each experiment was performed independently for three times at least.

### Western Blotting

Proteins from total cell lysates were resolved by SDS-PAGE, transferred to polyvinylidene fluoride membrane, blocked in 5% non-fat milk in TBST (Tris buffered saline with Tween 20), and blotted with the antibodies for HA (sc-7392, Santa Cruz), c-Myc (sc-40, Santa Cruz), P50 (sc-8414, Santa Cruz), P65 (#4764, Cell Signaling), p-P65 (#3033s, Cell Signaling), IκBα (#9242, Cell Signaling), p-IκBα (ab133462, Abcam), and β-actin (sc-47778, Santa Cruz). Each experiment was performed independently for three times at least.

### Co-Immunoprecipitation (Co-IP)

293T cells were co-transfected with RBP-Jκ vectors (with HA tag) and (wt or mt) Notch2 vectors (with myc tag) by Lipofectamine 2000 (Invitrogen). After 48 h, the harvested cells were lysed on ice for 1 h in lysis buffer (P0013, Beyotime) with 1 mM phenylmethanesulfonyl fluoride. Co-IP was performed using HA antibody (sc-7392, Santa Cruz), and co-precipitated proteins were collected using protein G Plus-Agarose Immunoprecipitation Reagent (sc-2002, Santa Cruz), and then analyzed by western blotting.

### Proliferation Assay

Cell proliferation activity was assessed with the (3-(4,5-dimethylthiazol-2-yl)-2,5-diphenyl) tetrazolium bromide (MTT) assay essentially as described [33]. Lymphoma cells were seeded into 96-well plates at a density of 2×10^4^ cells/well, then the proliferation rates were measured at 24 h, 48 h, 72 h and 96 h. Absorbance values were measured at 570 nm with a microplate reader. Stably-infected Pfeiffer cell lines (wt Notch2, mt Notch2 and pLVX cells) were seeded into 96-well plates at a density of 3×10^4^ cells/well, treated with 25 µM PDTC (S1808, Beyotime) or DMSO for 24–72 h, the proliferation rates were also detected as described above. Each experiment was performed independently for three times at least.

### Statistical Analysis

Data was analyzed using Student's t-test to compare the results among wt Notch2 group, mt Notch2 group and pLVX group (empty vector group). A *P*-value of <0.05 was considered to be statistically significant.

## Results

### *NOTCH2* gene is mutated in a group of DLBCL cases

Exon 26 (HD domain) and exon 34 (PEST domain) of *NOTCH2* were extracted from the 115 B-NHL samples and amplified, then sequenced and compared with the germline sequences. The identical mutation of the three DLBCLs located at the nucleotide 7605 (G/A) in the human *NOTCH2* cDNA sequence (NM_024408.3), which was in the PEST domain and converted a tryptophan (DNA code: TGG) into a termination codon (DNA code: TGA). The mutant Notch2 protein lacked a part of the PEST domain, and terminated at 2435^th^ amino acids with lactamine at the C-terminal (Table 1). This mutation did not exist in the surrounding non-tumor tissues in No. 505 and No. 646 (Fig. 1B). The non-tumor tissue was not available in No. 445. The G7605A change is not listed in the public SNP database (http://www.ncbi.nlm.nih.gov/projects/SNP/). In our samples, the *NOTCH2* mutation rate was about 4.3% (3/69) in DLBCLs. Apart from DLBCLs, no *NOTCH2* mutation was detected in other subtypes either in the exon 26 or the exon 34 (Table S1).

**Table 1:** *NOTCH2* mutational status in three DLBCL cases.

Case	Sex	Age	Diagnosis	Nucleic acid change	Amino acid change	Immunohistochemistry						
						CD10	Bcl6	MUM1	Notch2	P65	P50	Ki67
445	Male	30	DLBCL under Femur Fascia	7605 G/A	2435 stop	−	+	+	+	+	+	+
505	Male	23	Small Intestine DLBCL	7605 G/A	2435 stop	+	+	+	+	+	+	+
646	Male	NA	Small Intestine DLBCL	7605 G/A	2435 stop	+	+	+	+	+	+	+

Abbreviation: NA, information not available; DLBCL, diffuse large B-cell lymphoma.

### The DLBCL cases with *NOTCH2* mutation show similar expression pattern

**Figure 2:**
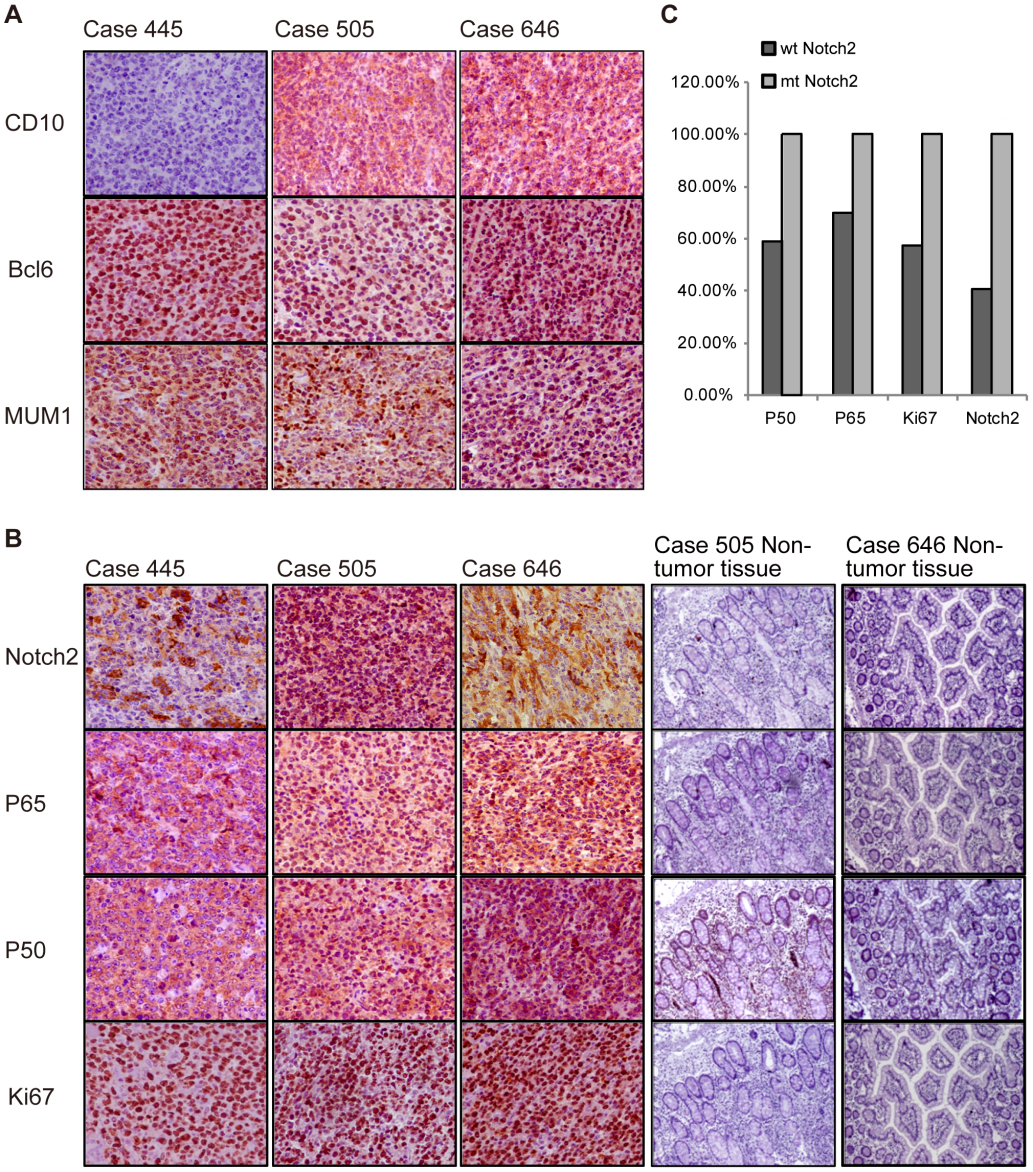
The immunohistochemical pattern of DLBCLs with the *NOTCH2* mutation. (A) The immunohistochemistry was used to analyze the immunohistochemical pattern of *NOTCH2* mutant DLBCLs with CD10, BCL6 and MUM-1 (×400 objective magnifications). (B) The immunohistochemistry was used to analyze the *NOTCH2* mutant DLBCLs (×400 objective magnifications) and the matched surrounding non-tumor tissues (×100 objective magnifications) with Notch2, P65, P50 and Ki67. But matched non-tumor tissue was not available in No. 445. (C) The positive rates of P50, P65, Ki67 and Notch2 were calculated in the two groups of DLBCLs (wt Notch2 and mt Notch2).

The three DLBCL cases with *NOTCH2* mutation all expressed BCL6 and MUM-1, but one case did not express CD10 (Fig. 2A), differed from previous report [18]. Among them, one (No. 445) was non-GCB DLBCL, and two (No. 505 and No. 646) were GCB DLBCL, according to Han's algorithm [28]. All the three *NOTCH2* mutated cases expressed Notch2, P65 and P50 (Fig. 2B), whereas Notch2 and P65 were both negative in their matched surrounding non-tumor tissues (Fig. 2B). And the strong positivity of Ki67 suggested a higher proliferation rate in *NOTCH2* mutant DLBCLs compared to the paired non-tumor tissues (Fig. 2B). Besides, the positive proportions of P50, P65, Ki67 and Notch2 in the DLBCLs with the wild-type *NOTCH2* were 59.09% (39/66), 69.70% (46/66), 57.58% (38/66) and 40.91% (27/66), respectively, which were significantly lower than those in *NOTCH2* mutated DLBCLs (Fig. 2C). Altogether, DLBCL cases carrying truncate Notch2 show similar immunohistochemical pattern, and they may exhibit stronger cell proliferation signals.

### The truncate Notch2 enhances the cell proliferation

**Figure 3:**
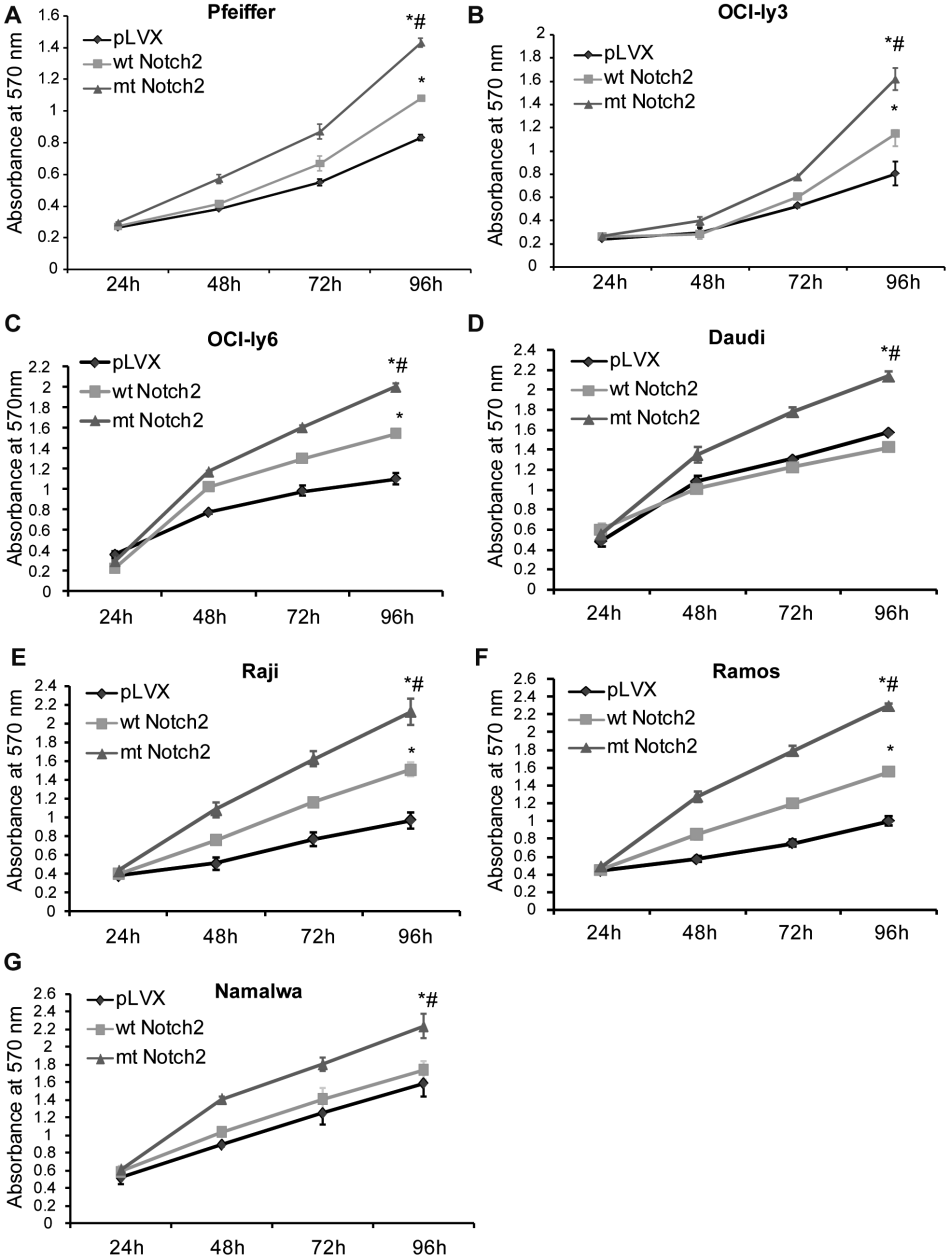
The truncate Notch2 enhances the cell proliferation. (A–G) MTT assay was used to detect the growth rates of the stably-infected lymphoma cell lines (Daudi, Raji, Ramos, Namalwa, OCI-ly3, OCI-ly6 and Pfeiffer), including wt Notch2 cells, mt Notch2 cells and pLVX cells. The MTT detected the absorbance (570 nm) at 24 h, 48 h, 72 h and 96 h. *P<0.05 versus pLVX, while #*P*<0.05 versus wt Notch2. Each experiment was performed independently for three times at least.

MTT assay was used to assess the growth rates of the stably-infected DLBCL and Burkitt's lymphoma cell lines, and the results showed that mt Notch2 cells grew much faster than wt Notch2 cells and pLVX cells (Fig. 3). In the three DLBCL cell lines, wt Notch2 cells grew faster than pLVX cells; while in Burkitt's lymphoma cell lines, similar results were only found in Raji and Ramos cell lines (Fig. 3). These data suggest that the truncate Notch2 may own a stronger ability to trigger cell proliferation, compared with the wt Notch2.

### The truncate Notch2 receptor improves the activity of the Notch2 signaling

**Figure 4:**
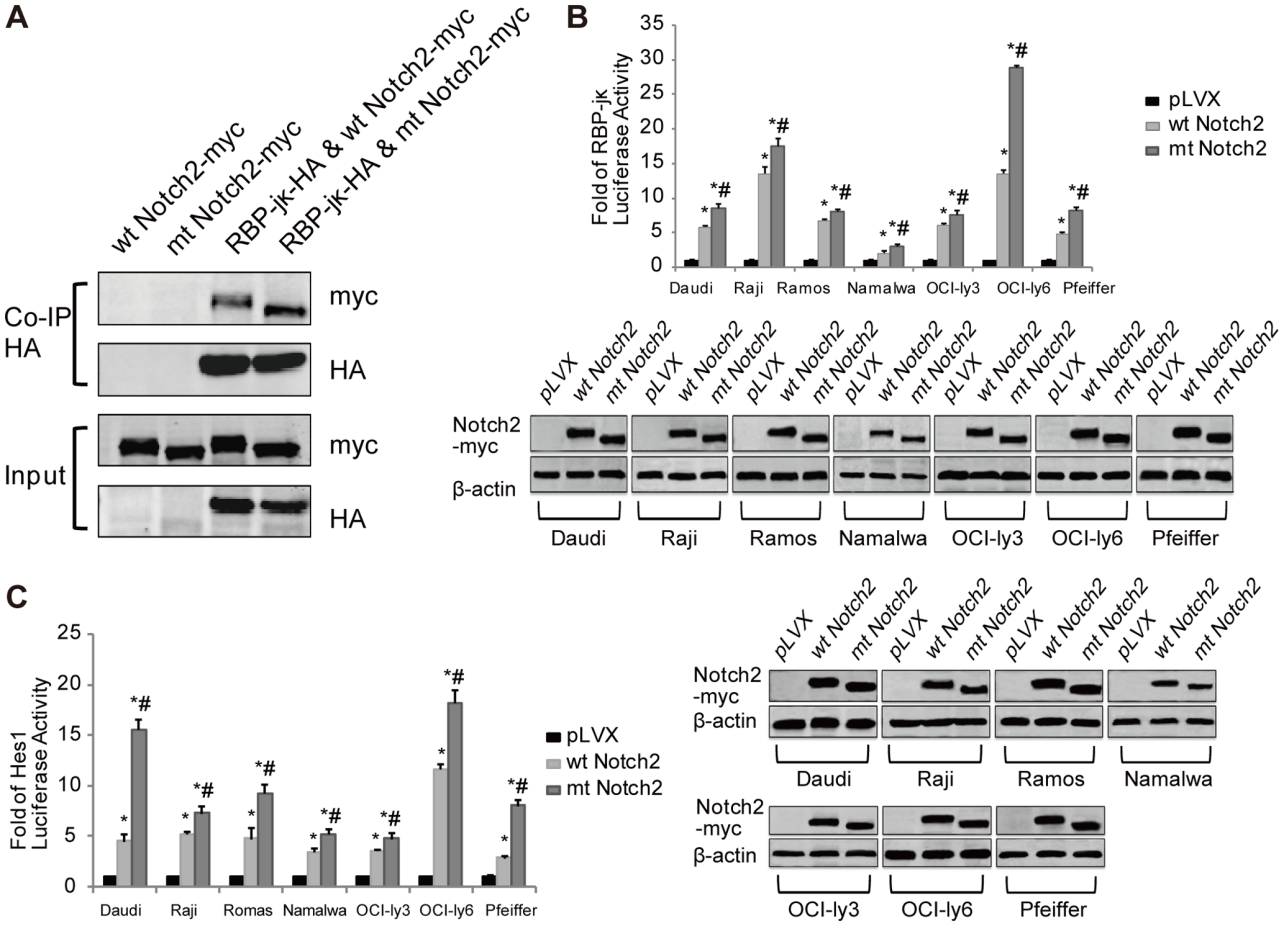
The truncate Notch2 receptor increases the activity of the Notch2 signaling. (A) Co-IP was used to analyze the binding ability between Notch2 (with c-myc tag) and RBP-jκ (with HA tag). (B) The RBP-Jκ reporter and (C) the Hes1 reporter were used to detect the luciferase activity of the lymphoma cell lines (Daudi, Raji, Ramos, Namalwa, OCI-ly3, OCI-ly6 and Pfeiffer) after transfection with the Notch2 (wt or mt) vectors or the pLVX vectors. *P<0.05 versus pLVX, while #*P*<0.05 versus wt Notch2. Each experiment was performed independently for three times at least.

The NICD cannot directly bind to DNA, but it can heterodimerize with the DNA-binding protein RBP-Jκ (an transcription factor) and activate the transcription of target genes containing RBP-Jκ binding sites [5], such as Hes1 [5], [6]. In the *in vivo* binding studies, the Co-IP results showed that mt Notch2 had stronger binding ability to RBP-Jκ than wt Notch2 (Fig. 4A). In the RBP-Jκ and the Hes1 luciferase reporter assays, the luciferase activities increased significantly in mt Notch2 cells, compared with those in wt Notch2 cells and pLVX cells (Fig. 4B–4C). These results indicated that mt Notch2 could significantly elevate the transcriptional activity of Notch2 signaling, compared with wt Notch2. Taken together, the truncate *NOTCH2* can promote the activation of the Notch2 signal pathway.

### The truncate Notch2 receptor activates the NF-κB signal pathway

**Figure 5:**
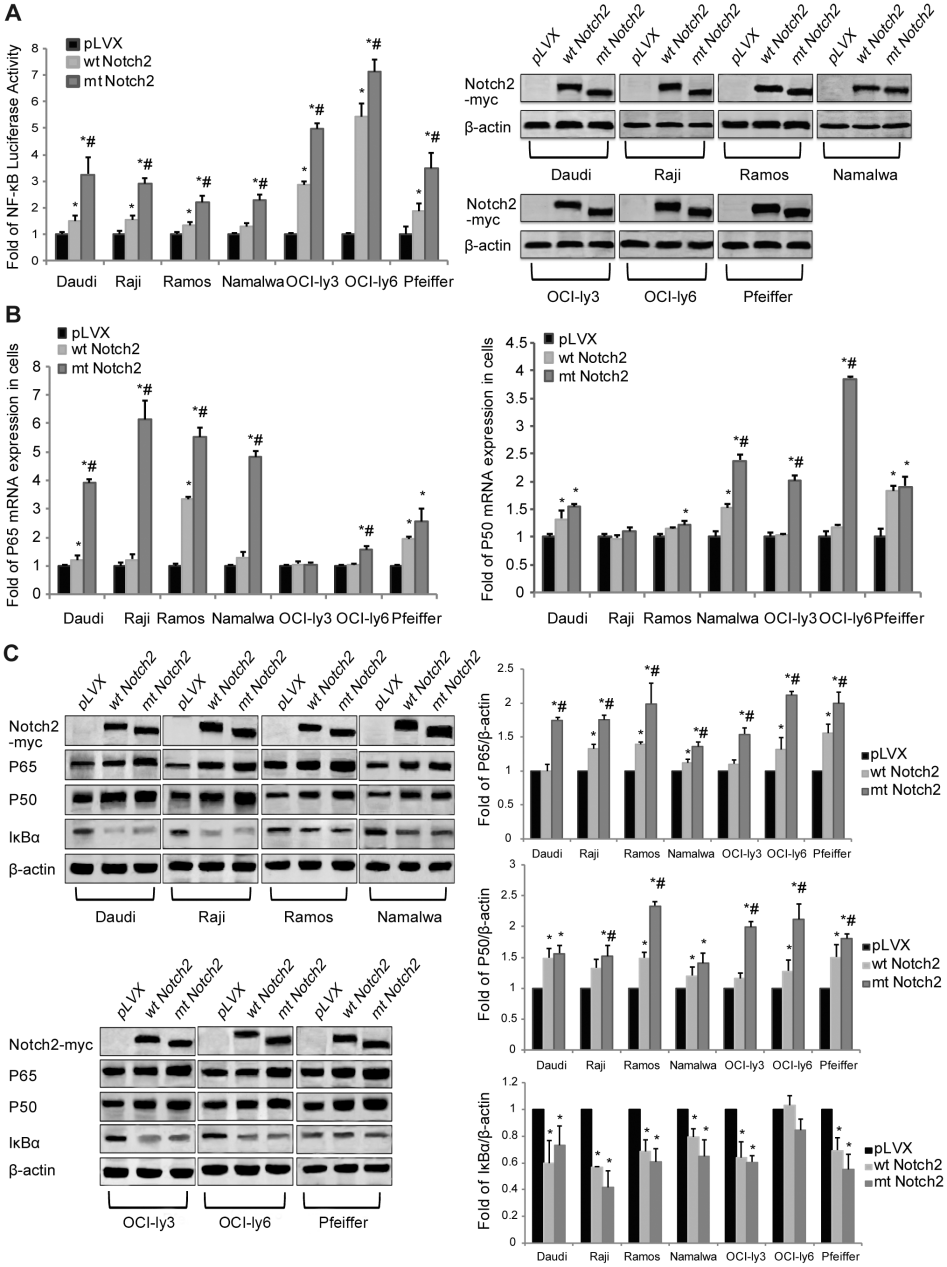
The truncate Notch2 activates the NF-κB signaling. (A) The luciferase activity of the NF-κB signaling and (B) real-time PCR of P50 and P65 in lymphoma cell lines (Daudi, Raji, Ramos, Namalwa, OCI-ly3, OCI-ly6 and Pfeiffer) were analyzed after transfection or infection with the Notch2 (wt or mt) or the pLVX vectors. (C) Western blotting and quantification of Notch2 (with c-myc tag), P65, P50, IκBα and β-actin were analyzed in the stably-infected lymphoma cell lines including wt Notch2 cells, mt Notch2 cells and pLVX cells. *P<0.05 versus pLVX, while #*P*<0.05 versus wt Notch2. Each experiment was performed independently for three times at least.

In this study, P50 and P65 were immunohistochemical positive in all mutant *NOTCH2* cases (Fig. 2B), so we tried to further explore the relationship of Notch2 with NF-κB in signal transfer. The NF-κB luciferase activity in mt Notch2 cells increased significantly in comparison to that in wt Notch2 cells and pLVX cells; and in wt Notch2 cells it was also higher than that in pLVX cells (Fig. 5A). Besides, the mRNA and protein levels of P65 were up-regulated obviously in mt Notch2 cells than pLVX cells and wt Notch2 cells; and these expression levels also rose in wt Notch2 cells (Fig. 5B–5C). Although the activation of truncate Notch2 on P50 was not as obvious as P65, the mRNA and protein levels of P50 in mt Notch2 DLBCL cells were up-regulated significantly in comparison to those in wt Notch2 cells and pLVX cells (Fig. 5B–5C). Moreover, in both wt Notch2 and mt Notch2 cells, the protein level of IκBα reduced compared with the pLVX cells (Fig. 5C). Altogether, these data indicate that the truncate Notch2 can activate the NF-κB signal pathway.

### The truncate Notch2 receptor enhances cell proliferation through up-regulating the phosphorylation levels of the NF-κB signal pathway

**Figure 6:**
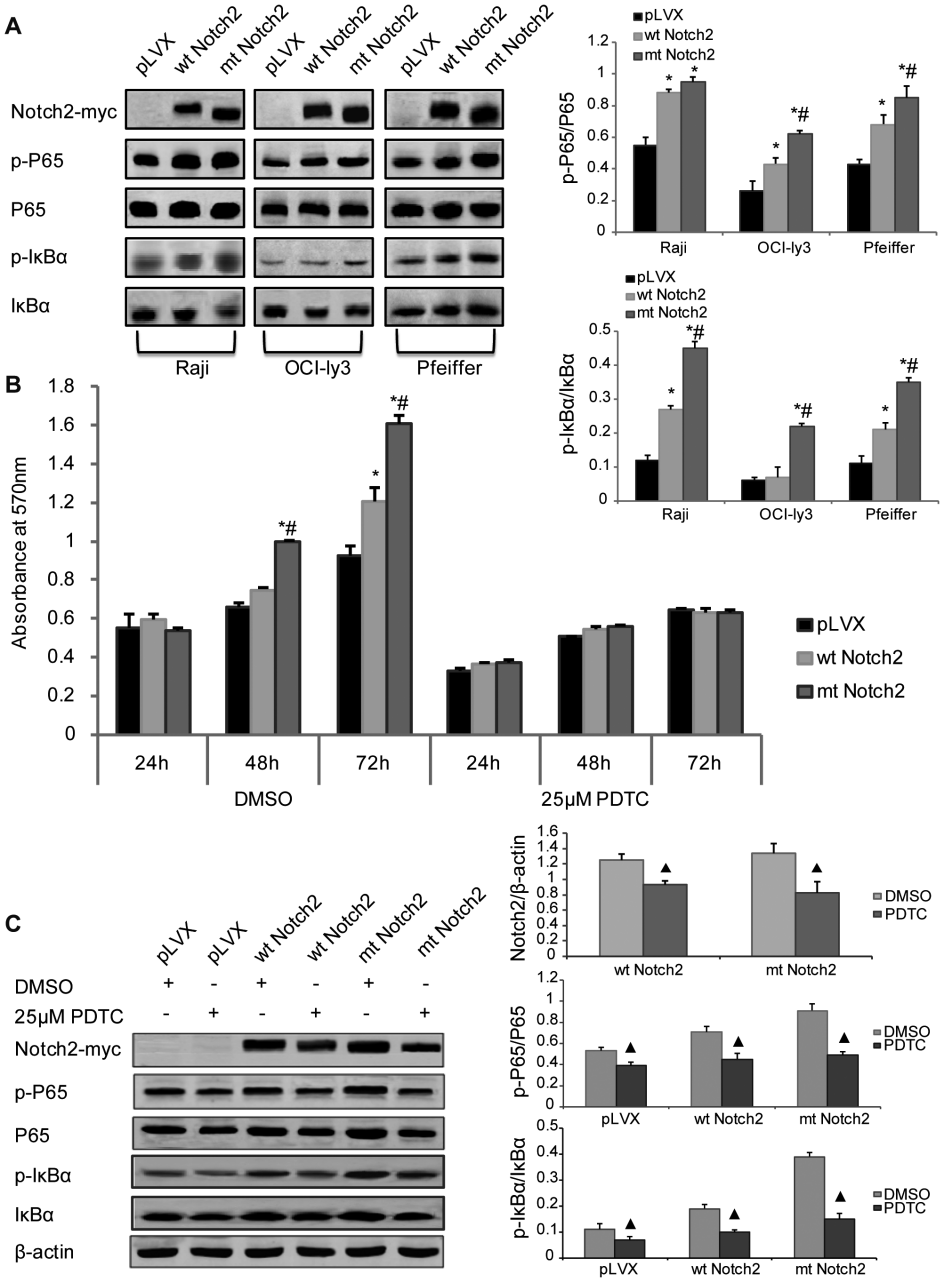
The truncate Notch2 enhances cell proliferation which may be associated with the phosphorylation levels of the NF-κB signaling. (A) Western blotting and quantification of Notch2 (with c-myc tag), P65, p-P65, IκBα and p-IκBα were analyzed in the stably-infected Raji cells, OCI-ly3 cells and Pfeiffer cells (including wt Notch2 cells, mt Notch2 cells and pLVX cells). (B) MTT assay was used to detect the growth rates of the stably-infected Pfeiffer cells after treatment with 25 µM PDTC for 24 h, 48 h and 72 h. (C) Western blotting and quantification of Notch2 (with c-myc tag), P65, p-P65, IκBα, p-IκBα and β-actin in the stably-infected Pfeiffer cells were analyzed after treatment with 25 µM PDTC or the same dose of DMSO for 48 h. *P<0.05 versus pLVX, #*P*<0.05 versus wt Notch2, ▴*P*<0.05 versus DMSO. Each experiment was performed independently for three times at least.

To further investigate the relationship between the Notch2 and the NF-κB signal pathways, we assayed the phosphorylation level of the NF-κB signal pathway. The western blotting results showed that in mt Notch2 DLBCL cells (including OCI-ly3 and Pfeiffer), the phosphorylation levels of P65 and IκBα were up-regulated significantly compared with those in wt Notch2 cells and pLVX cells, and in wt Notch2 DLBCL cells they were higher than pLVX cells (Fig. 6A). And similar results were found in Raji cells, but the phosphorylation level of P65 in wt Notch2 cells and mt Notch2 cells were similar (Fig. 6A). These results may imply that truncate Notch2 can activate the NF-κB signal pathway through up-regulating the phosphorylation levels of P65 and IκBα. Interestingly, Notch2 signaling was also affected by NF-κB signaling. PDTC (an inhibitor of NF-κB) was used to treat the stably-infected Pfeiffer cell lines (including wt Notch2 cells, mt Notch2 cells and pLVX cells). It could completely block the rapid proliferation of mt Notch2 cells, and there were no significant differences among mt Notch2 cells, wt Notch2 cells and pLVX cells (Fig. 6B). In addition, the protein expression levels of both wt Notch2 and mt Notch2 reduced after treatment with PDTC (Fig. 6C). These data suggest the truncate Notch2 improves cell proliferation may through up-regulating the phosphorylation level of NF-κB, and the Notch2 signaling is also affected by the NF-κB signal pathway.

## Discussion

Notch molecules are well conserved from Drosophila melanogaster to mammals [8], and the PEST domain is a hot mutation spot, as most *NOTCH2* mutations are clustered in this domain [18]–[20]. Moreover, the *NOTCH2* mutations identified in lymphoid malignancies (acute T-cell lymphocytic leukemia, chronic lymphocytic leukemia, SMZLs, and DLBCLs) are all gain-of-function mutations [18], [21], [34], [35]. In this study, we reported a recurring 7605 (G/A) mutation in the PEST domain of *NOTCH2* in 3 of 69 DLBCLs (4.3%). The mutation converted tryptophan (TGG) into a termination codon (TGA), thus deleting a part of the PEST domain and resulting in Notch2-reduced turnover, similarly to previous report [18]. Interestingly, our results showed the truncate *NOTCH2* could promote lymphoma cell proliferation and had stronger binding ability to RBP-Jκ, compared with the wild-type Notch2. RBP-Jκ is an important transcription factor of the Notch signaling, and it can interact with NICD through the RAM (RBP-Jκ associated molecule) and the ANK (six ankyrin/CDC10 repeats) domains, but not the PEST domain (Fig. 1A) [36]. However, losing a part of the PEST domain could prolong the half-life of NICD [37], as this domain is necessary for the ubiquitination-based degradation of NICD [38], [39]. As a result, the truncate Notch2 can interact with RBP-Jκ for a longer time, leading to higher activity of the Notch2 signaling. Altogether, we imply that Notch2 with PEST domain truncation can enhance cell proliferation through activating the Notch2 signaling, and this may be associated with DLBCL carcinogenesis.

DLBCL is the most common type of B-NHLs, and is well known to be highly heterogeneous clinically in both the western countries and China. All three DLBCLs with truncate Notch2 were immunohistochemically positive for Bcl6, MUM1, Notch2, P65, P50 and Ki67 concomitant with high rate of cell proliferation. Among these cases, two (No. 505 and No. 646) were rarely reported in DLBCLs, because of their tri-positive expression with CD10, BCL6 and MUM-1. Hans grouped these cases (about 6% in his report) into the GCB subtype rather than the non-GCB subtype [28]. In other studies, the DLBCLs (CD10+/BCL6+/MUM-1+) demonstrated “activated” GCB [40] or non-classified/non-GCB [29]. It remains controversial whether the DLBCLs (CD10+/BCL6+/MUM-1+) belong to the GCB group or the non-GCB group [40]. In our study, the cases with *NOTCH2* truncate mutation were positive for P65 and P50, thus they all had a characteristic of ABC DLBCLs, which was characterized by constitutive activation of the NF-κB signal pathway [41], [42].

The NF-κB signal pathway is well-known in controlling and maintaining cell viability through inhibition of apoptosis in response to environmental stress or cytotoxic agents [43]. Accumulating evidences have indicated that there was complex cross-talk between Notch and NF-κB [44], [45]: NF-κB2 is a Notch target gene [46], and NF-κB signaling increases the expression of Notch receptors and ligands [13], NICD can physically interact with NF-κB and compete with IκBα leading to retention of NF-κB in the nucleus [47]. Similarly to these findings, our present study showed that the truncate Notch2 could increase the activation of NF-κB through up-regulating its phosphorylation, total protein, mRNA and transcriptional levels; and PDTC (an NF-κB inhibitor) could also modulate the Notch2 signal pathway. Thus, we hypothesize that the enhancement of cell proliferation induced by the truncate Notch2 is associated with the activation of the NF-κB signal pathway.

The Notch pathway may be a new target for individual tumor treatment. Notch inhibitors, such as γ-secretase inhibitors (GSIs) that prevent Notch proteolytic cleavage into NICD, are already available and under clinical trials [48]–[50]. Three GSI compounds (GSI-I, IX, and XII) have been reported to induce significant apoptosis and suppress the growth of B-cell malignant lymphoma cell lines, including Burkitt's lymphoma cells and DLBCL cells [51], [52]. However, the use of GSIs faces several challenges, such as the toxicity and the resistance of GSIs [53]. As the resistance to single drug emerges rapidly, a multidrug chemotherapy targeting Notch and associated pathways is needed, such as NF-κB inhibitors [53]–[55].

In this study, we report a recurrent mutant *NOTCH2* site with PEST domain truncation in DLBCLs. The truncate Notch2 receptor promotes lymphoma cell proliferation which may be associated with the activation of both the Notch2 and the NF-κB signaling, and there may be a cross-talk between the Notch2 and the NF-κB signals. These findings suggest a new potential candidate target for treating DLBCLs by suppressing the Notch2 signal pathway alone or in combination with the NF-κB signal pathway.

## Supporting Information

Table S1 **The mutational status in non-Hodgkin's B cell lymphomas.** Abbreviations: DLBCL, diffuse large B cell lymphoma; MALT, mucosa-associated lymphoid tissue; SLL, small B cell lymphoma; FL, follicular lymphoma; Burkitt's, Burkitt's lymphoma. 115 non-Hodgkin's B cell lymphoma cases were collected from affiliated hospitals of Zhejiang University, and five of them have both DLBCL and MALT. 3 of 69 (4.3%) DLBCLs carried the *Notch2* mutations in the exon34, and no *NOTCH2* mutation was detected in other subtypes either in the exon 26 or the exon 34.
(DOCX)

Table S2 **The primer sequences of Real-time PCR in this study.**
(DOCX)
