# Supplementary material for: The Truncate Mutation of Notch2 Enhances Cell Proliferation through Activating the NF-κB Signal Pathway in the Diffuse Large B-Cell Lymphomas
Source: PLoS One. 2014 Oct 14;9(10):e108747. doi: 10.1371/journal.pone.0108747 (PMC4196756; doi:10.1371/journal.pone.0108747)
Supplement: Table S1 — The mutational status in non-Hodgkin's B cell lymphomas. Abbreviations: DLBCL, diffuse large B cell lymphoma; MALT, mucosa-associated lymphoid tissue; SLL, small B cell lymphoma; FL, follicular lymphoma; Burkitt's, Burkitt's lymphoma. 115 non-Hodgkin's B cell lymphoma cases were collected from affiliated hospitals of Zhejiang University, and five of them have both DLBCL and MALT. 3 of 69 (4.3%) DLBCLs carried the Notch2 mutations in the exon34, and no NOTCH2 mutation was detected in other subtypes either in the exon 26 or the exon 34. (DOCX) [file pone.0108747.s001.docx]

Table S1: The mutational status in non-Hodgkin’s B cell lymphomas

|  | DLBCL | MALT | FL | SLL | Burkitt’s | undetermined |
| --- | --- | --- | --- | --- | --- | --- |
| Number | 69 | 25 | 10 | 8 | 1 | 7 |
| Mutations in exon34 | 3 | 0 | 0 | 0 | 0 | 0 |
| Mutation Rate (%) | 4.3 | 0 | 0 | 0 | 0 | 0 |

Abbreviations: DLBCL, diffuse large B cell lymphoma; MALT, mucosa-associated lymphoid tissue; SLL, small B cell lymphoma; FL, follicular lymphoma; Burkitt’s, Burkitt’s lymphoma. 115 non-Hodgkin’s B cell lymphoma cases were collected from affiliated hospitals of Zhejiang University, and five of them have both DLBCL and MALT. 3 of 69 (4.3%) DLBCLs carried the *NOTCH2* mutations in the exon34, and no *NOTCH2* mutation was detected in other subtypes either in the exon 26 or the exon 34.
