# Supplementary material for: The Truncate Mutation of Notch2 Enhances Cell Proliferation through Activating the NF-κB Signal Pathway in the Diffuse Large B-Cell Lymphomas
Source: PLoS One. 2014 Oct 14;9(10):e108747. doi: 10.1371/journal.pone.0108747 (PMC4196756; doi:10.1371/journal.pone.0108747)
Supplement: Table S2 — The primer sequences of Real-time PCR in this study. (DOCX) [file pone.0108747.s002.docx]

| Table S2: The primer sequences of Real-time PCR | |
| --- | --- |
| Name | Sequence |
| Notch2-F | CTGGATGCAGGTGCAGATGCCAATGC |
| Notch2-R | GCAGAAGTCAACACGGTGCCTGGAGG |
| P50-F | ATCTGTACCAGACGCCCTTG |
| P50-R | AGTGCTGCCTTTTTGTGCT |
| P65-F | TGCCGAGTGAACCGAAAC |
| P65-R | TGGTGCTCAGGGATGACG |
| GAPDH-F | CAACGGATTTGGTCGTATTGG |
| GAPDH-R | CTGGAAGATGGTGATGGGATT |
